# On Gray Images of Cyclic and Self-Orthogonal Codes over $F_{q}+uF_{q}+vF_{q}$

**DOI:** 10.3390/e28020250

**Published:** 2026-02-22

**Authors:** Sami H. Saif, Alhanouf Ali Alhomaidhi

**Affiliations:** Department of Mathematics, College of Science, King Saud University, P.O. Box 2455, Riyadh 11451, Saudi Arabia

**Keywords:** non-Frobenius rings, Gray maps, cyclic codes, quasi-cyclic codes, code duality, 11T71, 94B15, 94B05

## Abstract

Let *p* be a prime with p∉{2,5} and let $q=p^{m}$. This paper studies cyclic and self-orthogonal linear codes of length *n* over the finite local non-Frobenius ring $R_{p,u,v}=F_{q}+uF_{q}+vF_{q},\;u^{2}=v^{2}=uv=vu=0.$ We define an $F_{q}$-linear Gray map $\pi_{n}:R_{p,u,v}^{n}\to F_{q}^{6n}$ and investigate the structural properties of Gray images of cyclic codes under this map. It is shown that $\pi_{n}$ preserves self-orthogonality and, when gcd(n,p)=1, transforms any cyclic code over $R_{p,u,v}$ into a quasi-cyclic code over $F_{q}$ of length 6n with index dividing 6. Moreover, we completely characterize the possible quasi-cyclic indices of the Gray images, proving that only the values l∈{1,3,6} can occur, and we establish necessary and sufficient conditions for each case in terms of the generators of the associated cyclic code. Several explicit examples are provided to illustrate the theoretical results and the resulting quasi-cyclic structures.

## 1. Introduction

Cyclic codes have been a central object of study in algebraic coding theory for several decades due to their strong algebraic structure and efficient implementation in communication and storage systems [1]. Their invariance under cyclic shifts allows a polynomial representation that facilitates both structural analysis and decoding. Quasi-cyclic codes arise as a natural generalization by requiring invariance under a fixed power of the shift operator, and they form a broad class of linear codes containing many families with good algebraic and combinatorial properties [2,3,4]. In particular, several important subclasses of linear codes, including constacyclic and linear complementary dual (LCD) codes, naturally embed within the quasi-cyclic framework [5,6,7,8].

The interaction between ring theory and coding theory gained significant momentum with the seminal work of Hammons et al. [9], who demonstrated that several classical nonlinear binary codes can be realized as Gray images of linear codes over $Z_{4}$. This discovery initiated extensive research on linear codes over finite rings and on Gray maps as structure-preserving transformations linking codes over rings to codes over finite fields. Subsequent studies examined cyclic and constacyclic codes over rings such as $Z_{p^{k}}$ and $Z_{p^{2}}$, with particular emphasis on determining when Gray images preserve cyclic or quasi-cyclic structures [10,11,12].

The algebraic structure of quasi-cyclic codes over finite fields was further clarified by Ling and Solé [11], who developed a module-theoretic framework based on polynomial quotient rings and the Chinese Remainder Theorem. Their approach enabled systematic classification results and influenced a wide range of subsequent studies. These ideas were later extended to codes over finite chain rings and, more recently, to non-chain and non-Frobenius rings, leading to new families of cyclic and quasi-cyclic codes with additional algebraic constraints [13,14,15,16]. Despite this progress, the behavior of Gray images of cyclic codes over non-Frobenius rings remains significantly less understood than in classical chain-ring or Frobenius settings.

From a structural viewpoint, non-Frobenius rings are of particular interest because many of the standard tools used in coding theory, such as canonical duality, MacWilliams-type identities, and decomposition techniques, either fail to apply or behave fundamentally differently compared with the Frobenius or chain-ring cases [17,18]. As a result, algebraic properties such as cyclicity, quasi-cyclicity, and self-orthogonality cannot, in general, be transferred automatically from codes over the ring to their images over finite fields via Gray maps, even when the maps are linear and distance-preserving. Determining which algebraic invariances are preserved under such mappings and which are lost therefore becomes a genuine structural problem rather than a routine extension of known results, particularly in the setting of non-chain and non-Frobenius rings [19,20,21].

In this paper, we study cyclic codes over the finite commutative local non-Frobenius ring

(1) $$R_{p,u,v}=F_{q}+uF_{q}+vF_{q},\;u^{2}=v^{2}=uv=vu=0,$$

where $q=p^{m}$ and *p* is a prime. This ring is not a chain ring and does not admit a linear ordering of ideals, which leads to structural phenomena that do not arise in more classical settings. In particular, cyclic codes over $R_{p,u,v}$ may involve multiple interacting nilpotent components, making the analysis of their Gray images more delicate than in previously studied ring families.

The motivation for the present work is to obtain a precise structural understanding of how cyclic and self-orthogonal codes over $R_{p,u,v}$ behave under Gray mappings into vector spaces over finite fields. Gray maps have long been recognized as fundamental tools for relating codes over rings to classical linear codes over fields [9]. Building on this idea, we introduce an explicit $F_{q}$-linear Gray map from $R_{p,u,v}^{n}$ to $F_{q}^{6n}$ and study its interaction with cyclic shift operators and Euclidean duality. Under the natural assumption gcd(n,p)=1, we show that the Gray image of any cyclic code over $R_{p,u,v}$ is necessarily a quasi-cyclic code over $F_{q}$ with index dividing 6, extending known quasi-cyclic behavior of Gray images from chain-ring and related settings to the present non-Frobenius context [2,22].

A central result of this work is the complete classification of the quasi-cyclic indices that can arise from Gray images of cyclic codes over $R_{p,u,v}$. We prove that only the values 1, 3, and 6 are possible, and we establish necessary and sufficient conditions for each case in terms of the generators of the associated cyclic codes. In addition, we show that self-orthogonality is preserved under the proposed Gray map, despite the absence of the Frobenius property in the underlying ring. These results provide a unified structural description of the relationship between cyclic codes over $R_{p,u,v}$ and their Gray images over finite fields.

The remainder of the paper is organized as follows. In Section 2, we recall basic properties of the ring $R_{p,u,v}$ and introduce the Gray map used throughout the paper. Section 3 contains the main results concerning the quasi-cyclic structure and self-orthogonality of Gray images of cyclic codes, assuming gcd(n,p)=1. In Section 4, we present explicit examples and generator matrices illustrating the different quasi-cyclic behaviors predicted by the theoretical results.

## 2. Preliminaries

Let *p* be a prime number and let *m* be a positive integer. Set $q=p^{m}$ and denote by $F_{q}$ the finite field with *q* elements. Throughout this paper, we work over the finite commutative ring

(2) $$R_{p,u,v}=\frac{F_{q}\left[u,v\right]}{\langle u^{2},\;v^{2},\;uv,\;vu\rangle },$$

which can be identified with the $F_{q}$-algebra $F_{q}+uF_{q}+vF_{q}$, where the indeterminates satisfy $u^{2}=v^{2}=uv=vu=0$. The ring $R_{p,u,v}$ is local with unique maximal ideal

(3) J=〈u,v〉,

and it is not Frobenius. In particular, it does not admit the usual duality properties available for finite Frobenius or chain rings.

Let δ be a generator of the multiplicative group $F_{q}^{\times }$ of order q−1. The associated Teichmüller set is defined by

(4) T(m)=〈δ〉∪{0}.

Every element $z\in R_{p,u,v}$ admits a unique representation of the form

(5) z=f+ug+vh,f,g,h∈T(m).

An element *z* is a unit in $R_{p,u,v}$ if and only if its constant component *f* is nonzero in $F_{q}$. This simple characterization of units plays an important role in the algebraic description of codes over $R_{p,u,v}$.

A linear code C of length *n* over $R_{p,u,v}$ is an $R_{p,u,v}$-submodule of $R_{p,u,v}^{n}$. Codewords are written as *n*-tuples $\left(c_{0},c_{1},\ldots ,c_{n-1}\right)$ with $c_{i}\in R_{p,u,v}$. The cyclic shift operator on $R_{p,u,v}^{n}$ is defined by

(6) $$\sigma \left(c_{0},c_{1},\ldots ,c_{n-1}\right)=\left(c_{n-1},c_{0},\ldots ,c_{n-2}\right).$$

A code C is said to be cyclic if it is invariant under σ. More generally, for a positive integer *l*, a code of length nl over $R_{p,u,v}$ is called quasi-cyclic of index *l* if it is invariant under the *l*-fold shift $\sigma^{\left(l\right)}$. The case l=1 corresponds to classical cyclic codes.

There is a standard polynomial representation that identifies $R_{p,u,v}^{n}$ with the quotient module

$$ℜ\left(R_{p,u,v},n\right)=\frac{R_{p,u,v}\left[t\right]}{\langle t^{n}-1\rangle },$$

via the correspondence

$$\left(c_{0},c_{1},\ldots ,c_{n-1}\right)⟷c\left(t\right)=c_{0}+c_{1}t+\cdots +c_{n-1}t^{n-1}.$$

Under this identification, cyclic codes of length *n* over $R_{p,u,v}$ correspond precisely to ideals of $ℜ(R_{p,u,v},n)$, allowing the study of cyclic codes to be reduced to an ideal-theoretic problem in a polynomial quotient ring [23,24,25]. Duality also plays a central role in the study of codes over $R_{p,u,v}$. For vectors $a=(a_{0},\ldots ,a_{n-1})$ and $b=(b_{0},\ldots ,b_{n-1})$ in $R_{p,u,v}^{n}$, the Euclidean inner product is defined by

(7) $$a\cdot b=\sum_{i=0}^{n-1}a_{i}b_{i}.$$

The dual code of C is then given by

(8) $$C^{⊥}=\left\{d\in R_{p,u,v}^{n}|c\cdot d=0\;\mathrm{for}\;\mathrm{all}\;c\in C\right\}.$$

A code C is called self-orthogonal if $C\subseteq C^{⊥}$, and self-dual if $C=C^{⊥}$. These notions will be essential in the analysis of Gray images in subsequent sections.

## 3. Main Results

In this work, we fix *n* to be a positive integer such that gcd(p,n)=1, with the additional restriction that p∉{2,5}. The following result plays a central role in our investigation, as it provides a complete description of the algebraic structure of the ideals in the quotient ring

(9) $$ℜ=\frac{R_{p,u,v}\left[t\right]}{\langle t^{n}-1\rangle },$$

which in turn characterizes all cyclic codes of length *n* over $R_{p,u,v}$. We therefore begin by formally stating this fundamental outcome.

Since cyclic codes are a special case of constacyclic codes, the structural description established in (Theorem 1 in (p. 4, [2])) applies directly in our setting. Thus, we state the following result.

**Theorem** **1.** *Let $\hat{a}\left(t\right)=\frac{\left(t^{n}-1\right)}{a(t)}.$ A cyclic code C of length n over $R_{p,u,v}$ has a corresponding ideal of the form*

(10) I=〈a(t)+ub(t),vc(t)〉,

*where the polynomials satisfy*

(11) $$\begin{array}{cc} \; & c\left(t\right)|a\left(t\right)|t^{n}-1,\;\deg\left(b\right)<\deg\left(c\right)\;\mathrm{or}\;b\left(t\right)=0,\;c\left(t\right)|b\left(t\right)\hat{a}\left(t\right). \end{array}$$

We begin by recalling the notion and some fundamental properties of the quadratic character of a finite field. The quadratic character of the multiplicative group $F_{q}^{\times }$ is the function

(12) $$\eta :F_{q}^{\times }⟶\left\{\pm 1\right\},$$

defined by setting η(t)=1 if *t* is a nonzero square in $F_{q}^{\times }$ and η(t)=−1 if *t* is a nonsquare. For example, we clearly have η(1)=1, while η(δ)=−1, where δ denotes a fixed generator of $F_{q}^{\times }$. It is well known that

(13) η(−1)=−1ifandonlyifp≡3(mod4).

To extend this function to the entire field $F_{q}$, we adopt the convention η(0)=0. With this extension, the quadratic character admits the compact expression

(14) $$\eta \left(t\right)=t^{\frac{q-1}{2}}\;\mathrm{for}\;\mathrm{all}\;t\in F_{q}.$$

In the remainder of this work, the symbol η will consistently denote the quadratic character of $F_{q}$.

**Lemma** **1.** *Let $q=p^{m}$ with odd prime p. The number N of solutions $\left(\lambda_{1},\lambda_{2}\right)\in F_{q}^{2}$ to*

(15) $$\lambda_{1}^{2}+\lambda_{2}^{2}=-1$$

*is*

$$N\;=\;q-\eta \left(-1\right)\;=\;\left\{\begin{array}{cc} q-1, & \mathrm{if}\;-1\;\mathrm{is}\;a\;\mathrm{square}\;\mathrm{in}\;F_{q}\;\left(\mathrm{equivalently}\;q\equiv 1\;\;\;\left(\mathrm{mod}\;4\right)\right),\\ q+1, & \mathrm{if}\;-1\;\mathrm{is}\;a\;\mathrm{nonsquare}\;\mathrm{in}\;F_{q}\;\left(\mathrm{equivalently}\;q\equiv 3\;\;\;\left(\mathrm{mod}\;4\right)\right). \end{array}\right.$$

**Proof.**  Let $\psi :F_{q}\to C^{\times }$ be a fixed nontrivial additive character and η be the quadratic character on $F_{q}$ (extended by η(0)=0). Using the standard additive-character indicator,

(16) $$1_{\left\{z=0\right\}}=\frac{1}{q}\sum_{t\in F_{q}}\psi \left(tz\right),$$

we can write

$$N=\sum_{x,y\in F_{q}}1_{\left\{x^{2}+y^{2}+1=0\right\}}=\frac{1}{q}\sum_{t\in F_{q}}\psi \left(t\right)\;\left(\sum_{x\in F_{q}}\psi \left(tx^{2}\right)\right)\;\left(\sum_{y\in F_{q}}\psi \left(ty^{2}\right)\right).$$

For t=0, the inner sums are both *q*, giving the contribution *q*. For t≠0, the quadratic Gauss sum identity yields

$$\sum_{x\in F_{q}}\psi \left(tx^{2}\right)=\eta \left(t\right)G,\;G=\sum_{x\in F_{q}}\psi \left(x^{2}\right),$$

so the t≠0 terms contribute

$$\frac{1}{q}\sum_{t\neq 0}\psi \left(t\right)\;\eta {\left(t\right)}^{2}\;G^{2}=\frac{G^{2}}{q}\sum_{t\neq 0}\psi \left(t\right)=\frac{G^{2}}{q}\left(\sum_{t\in F_{q}}\psi \left(t\right)-1\right)=-\frac{G^{2}}{q},$$

because $\sum_{t\in F_{q}}\psi \left(t\right)=0$. The fundamental relation for quadratic Gauss sums states $G^{2}=\eta \left(-1\right)\;q$. Substituting this gives

(17) $$N\;=\;q-\frac{G^{2}}{q}\;=\;q-\eta \left(-1\right).$$

Finally, η(−1)=1 if and only if −1 is a square in $F_{q}$, which holds exactly when q≡1(mod4); otherwise, η(−1)=−1 and q≡3(mod4). This yields the stated cases. □

Given $f\left(t\right)\in ℜ\left(F_{q},n\right)$, we write $f\left(t\right)=\sum_{i=0}^{n-1}f_{i}t^{i},$ where $f_{i}\in F_{q}.$ Similarly, for $f\left(t\right),g\left(t\right)\in ℜ\left(F_{q},n\right)$, we have

(18) $$f\left(t\right)g\left(t\right)=\sum_{i=0}^{n-1}{\left(fg\right)}_{i}t^{i},$$

where ${\left(fg\right)}_{i}$ are elements of $F_{q}.$

**Definition** **1.** *Let p be a prime with p∉{2,5}, and let C be a cyclic code of length n over $R_{p,u,v}$. Fix $\lambda_{1},\lambda_{2}\in F_{q}^{\times }$ with $\lambda_{1}^{2}+\lambda_{2}^{2}=-1$.*

*(i)*  *Gray maps and Lee weight: For f,g,h∈T(m), we define*
(19) $$\begin{array}{cc} \; & \omega_{L}\left(f+ug+vh\right)=\omega_{H}\left(\lambda_{1}g,f+\lambda_{2}g,g,\lambda_{1}h,f+\lambda_{2}h,h\right);\;(\mathrm{Lee}\;\mathrm{weight}) \end{array}$$
(20) $$\begin{array}{cc} \; & \pi \left(f+ug+vh\right)=\left(\lambda_{1}g,f+\lambda_{2}g,g,\lambda_{1}h,f+\lambda_{2}h,h\right),\;(\mathrm{Gray}\;\mathrm{maps}) \end{array}$$
  *extended to $\pi_{n}:R_{p,u,v}^{n}\to F_{q}^{6n}$ componentwise.*
*(ii)*  *Matrix representation: For $f=\left(f_{0},\ldots ,f_{6n-1}\right)\in F_{q}^{6n}$,*
(21) $$M\left(z\right)={\left[f_{6i+j}\right]}_{\begin{array}{c} 0\leq i\leq n-1\\ 0\leq j\leq 5 \end{array}}=\left(\begin{array}{cccccc} f_{0} & f_{1} & f_{2} & f_{3} & f_{4} & f_{5}\\ f_{6} & f_{7} & f_{8} & f_{9} & f_{10} & f_{11}\\ \vdots & \vdots & \vdots & \vdots & \vdots & \vdots \\ f_{6(n-1)} & \cdots & \cdots & \cdots & \cdots & f_{6n-1} \end{array}\right).$$
*For $f\left(t\right),g\left(t\right),h\left(t\right)\in ℜ\left(F_{q},n\right)$:*
(22) $$\pi_{n}\left(f\left(t\right)+ug\left(t\right)+vh\left(t\right)\right)={\left[\lambda_{1}g_{i},f_{i}+\lambda_{2}g_{i},g_{i},\lambda_{1}h_{i},f_{i}+\lambda_{2}h_{i},h_{i}\right]}_{i=0}^{n-1}.$$
*(iii)*  *Shift map: For $M=\left(f_{i,j}\right)\in F_{q}^{n\times 6}$,*
(23) $$\sigma \left(M\right)=\left(f_{i,j}^{\sigma }\right),\;f_{i,j}^{\sigma }=\left\{\begin{array}{cc} f_{i-1,5} & \mathrm{if}\;j=0,i\geq 1,\\ f_{n-1,5} & \mathrm{if}\;j=0,i=0,\\ f_{i,j-1} & \mathrm{if}\;j\geq 1. \end{array}\right.$$
*For instance, if n=2, then we have*
$$\sigma \left(\left(\begin{array}{cccccc} f_{0,0} & f_{0,1} & f_{0,2} & f_{0,3} & f_{0,4} & f_{0,5}\\ f_{1,0} & f_{1,1} & f_{1,2} & f_{1,3} & f_{1,4} & f_{1,5} \end{array}\right)\right)=\left(\begin{array}{cccccc} f_{1,5} & f_{0,0} & f_{0,1} & f_{0,2} & f_{0,3} & f_{0,4}\\ f_{0,5} & f_{1,0} & f_{1,1} & f_{1,2} & f_{1,3} & f_{1,4} \end{array}\right).$$

**Remark** **1.** *The quadratic constraint $\lambda_{1}^{2}+\lambda_{2}^{2}=-1$ arises from the requirement that the Gray map $\pi_{n}$ be compatible with the Lee weight on $R_{p,u,v}$ and the Hamming weight on $F_{q}^{6n}$. In particular, this relation ensures that the Gray map preserves orthogonality and yields the desired quasi-cyclic structure of the image codes. The exclusion of p∈{2,5} guarantees that non-degenerate solutions $\lambda_{1},\lambda_{2}\in F_{q}^{\times }$ satisfying this quadratic constraint exist.*

**Theorem** **2.** *Let C be a self-orthogonal cyclic code over $R_{p,u,v}$ of length n. Then, its Gray image $\pi_{n}\left(C\right)$ is also self-orthogonal.*

**Proof.**  Suppose that C is a self-orthogonal cyclic code over $R_{p,u,v}$ of length *n*. By definition, this means $C\subseteq C^{⊥}$. We claim that

$$\pi_{n}\left(C^{⊥}\right)\subseteq \pi_{n}{\left(C\right)}^{⊥}.$$

Take arbitrary codewords $c_{1}\in C$ and $c_{2}\in C^{⊥}$. Each $c_{i}$ can be expressed in the form

$$c_{i}=a_{i}+b_{i}u+e_{i}v,\;a_{i},b_{i},e_{i}\in F_{q}^{n}.$$

Since $c_{1}\cdot c_{2}=0$, we obtain the orthogonality conditions

(24) $$b_{1}\cdot a_{2}+a_{1}\cdot b_{2}=0,\;b_{1}\cdot e_{2}+a_{1}\cdot e_{2}=0,\;e_{1}\cdot e_{2}=0.$$

Now, consider their Gray images under $\pi_{n}$. A direct computation yields

$$\begin{array}{cc} \pi_{n}\left(c_{1}\right)\cdot \pi_{n}\left(c_{2}\right)\; & =\lambda_{1}^{2}\;b_{1}\cdot b_{2}+\left(b_{1}\cdot a_{2}+a_{1}\cdot b_{2}\right)+\lambda_{2}\left(b_{1}\cdot b_{2}+a_{1}\cdot b_{2}\right)\\ \; & \;+\lambda_{2}^{2}\;b_{1}\cdot b_{2}+b_{1}\cdot b_{2}+\lambda_{1}^{2}\;e_{1}\cdot e_{2}+\left(b_{1}\cdot e_{2}+a_{1}\cdot e_{2}\right)\\ \; & \;+\lambda_{2}\left(b_{1}\cdot e_{2}+a_{1}\cdot e_{2}\right)+\lambda_{2}^{2}\;e_{1}\cdot e_{2}+e_{1}\cdot e_{2}. \end{array}$$

Using the relations in (24), this expression simplifies to

$$\pi_{n}\left(c_{1}\right)\cdot \pi_{n}\left(c_{2}\right)=0.$$

Hence, $\pi_{n}\left(c_{2}\right)\in \pi_{n}{\left(C\right)}^{⊥}$. Since $c_{2}\in C^{⊥}$ was arbitrary, we conclude

$$\pi_{n}\left(C\right)\subseteq \pi_{n}\left(C^{⊥}\right)\subseteq \pi_{n}{\left(C\right)}^{⊥},$$

which shows that $\pi_{n}\left(C\right)$ is self-orthogonal. □

**Remark** **2.** *In this work, our primary focus is on cyclic and self-orthogonal codes over $R_{p,u,v}$. Since $R_{p,u,v}$ is not a Frobenius ring, the theory of self-dual cyclic codes involves additional structural considerations beyond those required for self-orthogonality. Although Theorem 2 shows that the Gray map preserves self-orthogonality, the characterization of self-dual cyclic codes and their Gray images involves additional constraints and constitutes a direction for future research.*

**Lemma** **2.** *Let C be a cyclic code over $R_{p,u,v}$ of length n, and let $I\subseteq ℜ(R_{p,u,v},n)$ be the ideal corresponding to C, generated as in Theorem 1 by*

I=〈a(t)+ub(t),vc(t)〉.

*Let $\pi_{n}$ be the Gray map defined in Definition 1, with $\lambda_{1},\lambda_{2}\in F_{q}^{\times }$ satisfying $\lambda_{1}^{2}+\lambda_{2}^{2}=-1$ as in Lemma 1. Then, for any f(t)∈I, there exist polynomials $f_{1}\left(t\right),f_{2}\left(t\right),f_{3}\left(t\right),g_{1}\left(t\right)\in ℜ\left(F_{q},n\right)$ such that*

$$f\left(t\right)=a\left(t\right)f_{1}\left(t\right)+u\left(b\left(t\right)f_{1}\left(t\right)+a\left(t\right)f_{2}\left(t\right)\right)+v\left(a\left(t\right)f_{3}\left(t\right)+c\left(t\right)g_{1}\left(t\right)\right).$$

*Moreover, the Gray image of f(t) can be expressed componentwise as*

(25) $$\pi_{n}\left(f\left(t\right)\right)={\left[f_{6i+j}\right]}_{\begin{array}{c} 0\leq i\leq n-1\\ 0\leq j\leq 5 \end{array}},$$

*where*

$$\begin{array}{cccc} f_{6i} & =\lambda_{1}\left({\left(bf_{1}\right)}_{i}+{\left(af_{2}\right)}_{i}\right), & f_{6i+1} & ={\left(af_{1}\right)}_{i}+\lambda_{2}\left({\left(bf_{1}\right)}_{i}+{\left(af_{2}\right)}_{i}\right),\\ f_{6i+2} & ={\left(bf_{1}\right)}_{i}+{\left(af_{2}\right)}_{i}, & f_{6i+3} & =\lambda_{1}\left({\left(af_{3}\right)}_{i}+{\left(cg_{1}\right)}_{i}\right),\\ f_{6i+4} & ={\left(af_{1}\right)}_{i}+\lambda_{2}\left({\left(af_{3}\right)}_{i}+{\left(cg_{1}\right)}_{i}\right), & f_{6i+5} & ={\left(af_{3}\right)}_{i}+{\left(cg_{1}\right)}_{i}. \end{array}$$

**Proof.**  Let f(t)∈I be arbitrary. By definition of *I*, it can be expressed in the form

$$f\left(t\right)=\left(a\left(t\right)+ub\left(t\right)+vc\left(t\right)\right)\left(f_{1}\left(t\right)+uf_{2}\left(t\right)+vf_{3}\left(t\right)\right)+vc\left(t\right)g_{1}\left(t\right),$$

for some polynomials $f_{1}\left(t\right),f_{2}\left(t\right),f_{3}\left(t\right),g_{1}\left(t\right)\in ℜ\left(F_{q},n\right)$. Using the relations $u^{2}=v^{2}=uv=vu=0$ in $R_{p,u,v}$, the above expression simplifies to

$$f\left(t\right)=a\left(t\right)f_{1}\left(t\right)+u\left(b\left(t\right)f_{1}\left(t\right)+a\left(t\right)f_{2}\left(t\right)\right)+v\left(a\left(t\right)f_{3}\left(t\right)+c\left(t\right)g_{1}\left(t\right)\right),$$

as claimed.

The componentwise formula for $\pi_{n}\left(f\left(t\right)\right)$ follows directly from the definition of the Gray map (Definition 1) and the choice of $\lambda_{1},\lambda_{2}$ as in Lemma 1. □

Next, we establish that under appropriate conditions on the parameters *n* and *p*, the Gray image of a cyclic code over $R_{p,u,v}$ of length *n* under the map $\pi_{n}$ possesses a quasi-cyclic structure. Specifically, we show that $\pi_{n}\left(C\right)$ is a quasi-cyclic code of length 6n over $F_{q}$ with a well-defined index, thereby extending the cyclicity properties of C in the context of its Gray image.

**Lemma** **3.** *Let C be a cyclic code over $R_{p,u,v}$ of length n, and let*

$$I=\langle a\left(t\right)+ub\left(t\right),\;vc\left(t\right)\rangle \subseteq ℜ\left(R_{p,u,v},n\right)$$

*be the ideal corresponding to C. Let $\pi_{n}$ be the Gray map defined in Definition 1, with $\lambda_{1},\lambda_{2}\in F_{q}^{\times }$ satisfying $\lambda_{1}^{2}+\lambda_{2}^{2}=-1$ as in Lemma 1. Then, the Gray image $\pi_{n}\left(C\right)$ is a quasi-cyclic code of length 6n over $F_{q}$ with index 6 at most.*

**Proof.**  Take any f(t)∈I and define g(t)=tf(t). Clearly, g(t)∈I since *I* is an ideal. By Lemma 1, the 6-fold cyclic shift of $\pi_{n}\left(f\left(t\right)\right)$ satisfies

$$\sigma^{\left(6\right)}\left(\pi_{n}\left(f\left(t\right)\right)\right)=\pi_{n}\left(g\left(t\right)\right).$$

Writing this componentwise, we have

$$\sigma^{\left(6\right)}\left(\pi_{n}\left(f\left(t\right)\right)\right)=\pi_{n}\left(g\left(t\right)\right)={\left[f_{6i+j}\right]}_{\begin{array}{c} 0\leq j\leq 5\\ i=0,1,\ldots ,n-1 \end{array}}.$$

This shows that $\pi_{n}\left(C\right)$ is invariant under the 6-fold cyclic shift, i.e., it is quasi-cyclic of index 6 at most. □

Under the assumptions of Lemma 2, we are now able to determine the precise index of the quasi-cyclic code $\pi_{n}\left(C\right)$. This allows us to characterize whether the Gray image of C is cyclic, quasi-cyclic of index 3, or quasi-cyclic of index 6, providing a complete classification of its quasi-cyclic structure.

**Theorem** **3.** *Let C be a cyclic code over $R_{p,u,v}$ of length n with I=〈a(t)+ub(t),vc(t)〉. Then, $\pi_{n}\left(C\right)$ is a quasi-cyclic code with index of either 1,3, or 6. Precisely, we have $\pi_{n}\left(C\right)$ is cyclic if and only if a(t)=c(t)=b(t)=0 and $\lambda_{1}=\lambda_{2}^{2},$ i.e., C=〈0〉.*

**Proof.**  By Lemma 3, $\pi_{n}\left(C\right)$ is a quasi-cyclic code of index 6 at most. Suppose that $\sigma^{\left(l\right)}\left(\pi_{n}\left(C\right)\right)=\pi_{n}\left(C\right)$ for some l≤6. Then, given f(t)∈I, there exists g(t)∈I such that $\sigma^{\left(l\right)}\left(\pi_{n}\left(f\left(t\right)\right)\right)=\pi_{n}\left(g\left(t\right)\right)$. In fact, whenever f(t) is an element of *I*, we always associate polynomials $f_{1}\left(t\right),f_{2}\left(t\right),f_{3}\left(t\right),g_{1}\left(t\right)$ of $ℜ(F_{q},n)$ such that $\pi_{n}\left(f\left(t\right)\right)=\left(f_{i,j}\right),$ where $f_{i,j}$ are elements of $F_{q}$ defined in Lemma 1, $f_{i,j}=f_{6i+j}.$ Similarly, when we choose an element g(t) of *I*, we always assume that there are polynomials $f_{1}^{\prime }\left(t\right),f_{2}^{\prime }\left(t\right),f_{3}^{\prime }\left(t\right),g_{1}^{\prime }\left(t\right)$ of $ℜ(F_{q},n)$ so that $\pi_{n}\left(g\left(t\right)\right)=M\left(g\left(t\right)\right)=\left(g_{ij}\right),$ where $g_{ij}=g_{6i+j}$ and

$$\begin{array}{cc} & g_{6i}=\lambda_{1}\left({\left(bf_{1}^{\prime }\right)}_{i}+{\left(ff_{2}^{\prime }\right)}_{i}\right),\;g_{6i+1}={\left(ff_{1}^{\prime }\right)}_{i}+\lambda_{2}\left({\left(bf_{1}^{\prime }\right)}_{i}+{\left(ff_{2}^{\prime }\right)}_{i}\right),\;g_{6i+2}={\left(bf_{1}^{\prime }\right)}_{i}+{\left(ff_{2}^{\prime }\right)}_{i},\\ & g_{6i+3}={\left(ff_{3}^{\prime }\right)}_{i}+{\left(cg_{1}^{\prime }\right)}_{i}),\;g_{6i+4}={\left(ff_{1}^{\prime }\right)}_{i}+{\left(ff_{3}^{\prime }\right)}_{i}+{\left(cg_{1}^{\prime }\right)}_{i}),\;g_{6i+5}={\left(ff_{3}^{\prime }\right)}_{i}+{\left(f_{2}g_{1}^{\prime }\right)}_{i}, \end{array}$$

for each i∈{0,…,n−1}. We may exclude the trivial case f(t)=0.

First, we prove that if c(t)≠0, then $\pi_{n}\left(C\right)$ has index 6. Suppose that $f_{2}\left(t\right)\neq 0$, and $\sigma^{\left(l\right)}\left(\pi_{n}\left(C\right)\right)=\pi_{n}\left(C\right)$ for some l≤5. Choose f(t)∈I. If l=1, then $\sigma \left(\pi_{n}\left(f\left(t\right)\right)\right)=\pi_{n}\left(g\left(t\right)\right)$ for some g(t)∈I. It follows that

$$\sigma \left(\pi_{n}\left(f\left(t\right)\right)\right)={\left[f_{6i+j}^{\sigma }\right]}_{\begin{array}{c} 0\leq i\leq n-1\\ 0\leq j\leq 5 \end{array}}=\pi_{n}\left(g\left(t\right)\right)={\left[g_{6i+j}\right]}_{\begin{array}{c} 0\leq i\leq n-1\\ 0\leq j\leq 5 \end{array}}.$$

In particular, $f_{6n-1}=g_{0}$ and $f_{1}=g_{2}$. So, by Lemma 2,

(26) $${\left(bf_{1}\right)}_{n-1}+{\left(ff_{3}\right)}_{n-1}+{\left(cg_{1}\right)}_{n-1}=\lambda_{1}\left({\left(bf_{1}^{\prime }\right)}_{0}+{\left(ff_{2}^{\prime }\right)}_{0}\right),$$

(27) $${\left(ff_{1}\right)}_{0}+\lambda_{2}\left({\left(bf_{1}\right)}_{0}+{\left(ff_{2}\right)}_{0}\right)={\left(bf_{1}^{\prime }\right)}_{0}+{\left(ff_{2}^{\prime }\right)}_{0}.$$

Multiplying $\lambda_{1}$ to both sides of Equation (27) and substituting Equation (26), we have

(28) $${\left(bf_{1}\right)}_{n-1}+{\left(ff_{3}\right)}_{n-1}+{\left(cg_{1}\right)}_{n-1}=\lambda_{1}\left({\left(ff_{1}\right)}_{0}+\lambda_{2}\left({\left(bf_{1}\right)}_{0}+{\left(ff_{2}\right)}_{0}\right)\right).$$

Note that this equation holds for arbitrary element f(t) of *I*. Hence, by setting $f_{1}\left(t\right)=f_{2}\left(t\right)=f_{3}\left(t\right)=0$ and $f_{1}\left(t\right)=t^{n-1-\deg(c(t))}$, we have ${\left(cg_{1}\right)}_{n-1}\neq 0$, but ${\left(bf_{1}\right)}_{n-1}={\left(ff_{3}\right)}_{n-1}={\left(ff_{1}\right)}_{0}={\left(bf_{1}\right)}_{0}={\left(ff_{2}\right)}_{0}=0$, which is a contradiction. The case when l∈{2,3,4,5} follows similarly. Hence, by Lemma 2, C has index 6, and the claim is proven.

Now, suppose that C satisfies the conditions given in the hypothesis. Then, it is clear that $\pi_{n}\left(C\right)$ is cyclic. Conversely, suppose that $\pi_{n}\left(C\right)$ is cyclic. Then, given f(t)∈I, there exists g(t)∈I such that $\sigma \left(\pi_{n}\left(f\left(t\right)\right)\right)=\pi_{n}\left(g\left(t\right)\right)$. So, we have

(29) $$\sigma \left(\pi_{n}\left(f\left(t\right)\right)\right)={\left[{\left(cg_{1}\right)}_{i},\lambda_{1}{\left(bf_{1}\right)}_{j},\lambda_{2}{\left(bf_{1}\right)}_{j},{\left(bf_{1}\right)}_{j},\lambda_{1}{\left(cg_{1}\right)}_{j},\lambda_{2}{\left(cg_{1}\right)}_{j}\right]}_{i=0}^{n-1}$$

while

(30) $$\pi_{n}\left(g\left(t\right)\right)={\left[\lambda_{1}{\left(bf_{1}^{\prime }\right)}_{j},\lambda_{2}{\left(bf_{1}^{\prime }\right)}_{j},{\left(bf_{1}^{\prime }\right)}_{j},\lambda_{1}{\left(cg_{1}^{\prime }\right)}_{j},\lambda_{2}{\left(cg_{1}^{\prime }\right)}_{j},{\left(cg_{1}^{\prime }\right)}_{i}\right]}_{i=0}^{n-1}.$$

Given i∈{0,…,n−1}, when comparing the (i,0)th, (i,2)th, (i,3)th, and (i,5)th entries of both matrices,

(31) $$\begin{array}{cc} {\left(cg_{1}\right)}_{i} & =\lambda_{1}{\left(bf_{1}^{\prime }\right)}_{i},\;\lambda_{2}{\left(bf_{1}\right)}_{i}={\left(bf_{1}^{\prime }\right)}_{i},\\ {\left(bf_{1}\right)}_{i} & =\lambda_{1}{\left(cg_{1}^{\prime }\right)}_{i},\;\lambda_{2}{\left(cg_{1}\right)}_{i}={\left(cg_{1}^{\prime }\right)}_{i}. \end{array}$$

(We write ${\left(cg_{1}\right)}_{-1}={\left(cg_{1}\right)}_{n-1}$ by convention). Multiplying $\lambda_{1}$, $\lambda_{1}\lambda_{2}$, and $\lambda_{1}^{2}\lambda_{2}$ to both sides of the equations in (31), respectively, and comparing with previous equations, we obtain

(32) $${\left(cg_{1}\right)}_{i-1}=\lambda_{1}^{2}\lambda_{2}^{2}{\left(cg_{1}\right)}_{i}\;\mathrm{for}\;\mathrm{each}\;i\in \left\{0,\ldots ,n-1\right\}.$$

On the other hand, by multiplying $\lambda_{1}$ to both sides of the last equation in (31) and substituting, we have

(33) $${\left(bf_{1}\right)}_{i}=\lambda_{1}\lambda_{2}{\left(cf_{1}\right)}_{i}\;\mathrm{for}\;\mathrm{each}\;i\in \left\{0,\ldots ,n-1\right\}.$$

Combining these two sets of equations, we obtain

(34) $${\left(bf_{1}\right)}_{i-1}=\lambda_{1}^{2}\lambda_{2}^{2}{\left(bf_{1}\right)}_{i}\;\mathrm{for}\;\mathrm{each}\;i\in \left\{0,\ldots ,n-1\right\}.$$

It also follows that, if ${\left(bf_{1}\right)}_{i}=0$ for some *i*, then ${\left(bf_{1}\right)}_{i}={\left(cg_{1}\right)}_{i}=0$ for all i∈{0,…,n−1}, so we have f(t)=0 by Lemma 2. Hence, we may assume that ${\left(bf_{1}\right)}_{i}\neq 0$ for each i∈{0,…,n−1}. Comparing the (i,1)th entries of $\sigma (\pi_{n}\left(f\left(t\right)\right))$ and $\pi_{n}\left(g\left(t\right)\right)$, we have $\lambda_{1}{\left(bf_{1}\right)}_{i}=\lambda_{2}{\left(bf_{1}^{\prime }\right)}_{i}.$ Multiplying $\lambda_{2}$ to both sides of (15) and substituting (17), we obtain $\lambda_{1}{\left(bf_{1}\right)}_{i}=\lambda_{2}^{2}{\left(bf_{1}\right)}_{i}$ and thus $\lambda_{1}=\lambda_{2}^{2}$. Since $\lambda_{1}^{2}+\lambda_{2}^{2}+1=0$ from our choice of $\lambda_{1}$ and $\lambda_{2}$, it follows that $\lambda_{1}^{2}+\lambda_{1}+1=0$. If $\lambda_{1}=1$, then p=3. Suppose that $\lambda_{1}\neq 1$. Then, $\lambda_{1}^{3}-1=\left(\lambda_{1}-1\right)\left(\lambda_{1}^{2}+\lambda_{1}+1\right)=0,$ so $\left\{1,\lambda_{1},\lambda_{1}^{2}\right\}$ is a cyclic subgroup of a cyclic group $F_{q}^{\times }$, the multiplicative group of units of $F_{q}$. It follows that the order of $F_{q}^{\times }$ must be a multiple of 3, which happens exactly when p≡1(mod3). On the other hand, $\lambda_{1}^{2}\lambda_{2}^{2}=\lambda_{1}^{3}=1$, so ${\left(bf_{1}\right)}_{0}={\left(bf_{1}\right)}_{1}=\cdots ={\left(bf_{1}\right)}_{n-1}$ and ${\left(cg_{1}\right)}_{0}={\left(cg_{1}\right)}_{1}=\cdots ={\left(cg_{1}\right)}_{n-1}$. Hence, $c\left(t\right)f_{1}\left(t\right)={\left(cf_{1}\right)}_{0}\left(1+\cdots +t^{n-1}\right)$ and $b\left(t\right)f_{1}\left(t\right)=\lambda_{1}\lambda_{2}c\left(t\right)g_{1}\left(t\right).$ Since this equation holds for arbitrary $f_{1}\left(t\right)\in ℜ\left(F_{q},n\right)$, by setting $f_{1}\left(t\right)=1$, we have $c\left(t\right)={\left(cg_{1}\right)}_{0}\left(1+\cdots +t^{n-1}\right)$ and $b\left(t\right)=\lambda_{1}\lambda_{2}c\left(t\right).$ This means that $C=\langle a\left(t\right)+ub\left(t\right),vc\left(t\right)\rangle =\langle a\left(t\right)+u\lambda_{1}\lambda_{2}c\left(t\right),vc\left(t\right)\rangle .$ By the claim stated above, c(t) must be 0, and it follows that C=〈a(t)〉=〈0〉 is cyclic. Hence, the result is proven. □

**Remark** **3.** *In light of the proof of Theorem 3, the results happen only if either p=3 or p≡1(mod3).*

**Remark** **4.** *Let C be a cyclic code of length n over $R_{p,u,v}$, and assume gcd(n,p)=1. Then, the Gray image $\pi_{n}\left(C\right)$ is a quasi-cyclic $F_{q}$-linear code of length 6n. Since the Gray map $\pi_{n}$ is $F_{q}$-linear and injective, it preserves the $F_{q}$-dimension. Hence, if $\dim_{F_{q}}\left(C\right)=k$, then $\pi_{n}\left(C\right)$ has length 6n and dimension k over $F_{q}$. Thus, the Gray image has parameters in the form [6n,k,d], where d denotes its minimum Hamming distance. Although the present work focuses primarily on the structural behavior of Gray images, particularly their quasi-cyclic index and preservation of orthogonality, a systematic investigation of minimum distance and optimality properties constitutes an interesting direction for future research.*

**Theorem** **4.** *Suppose that C be a cyclic code over $R_{p,u,v}$ of length n. Suppose also I=〈a(t)+ub(t),vc(t)〉. Then, $\pi_{n}\left(C\right)$ has index 3 if and only if $\pi_{n}\left(C\right)$ is not cyclic and c(t)=b(t)=0, that is, C=〈a(t)〉.*

**Proof.**  Suppose that l=3. We need to show that C satisfies conditions given in (ii). Given f(t)∈I, there exists g(t)∈I such that $\sigma^{\left(3\right)}\left(\pi_{n}\left(f\left(t\right)\right)\right)=\pi_{n}\left(g\left(t\right)\right).$ By the proof of Theorem 3, we have c(t)=0. We choose f(t)=a(t)+ub(t), then we obtain

$$\pi_{n}\left(f\left(t\right)\right)=\left[\begin{array}{cc} a & b \end{array}\right]\left[\begin{array}{cccccc} \lambda_{1} & \lambda_{2} & 1 & 0 & 0 & 0\\ 0 & 1 & 0 & 0 & 1 & 0 \end{array}\right],$$

where $a={\left(a_{0},a_{1},\ldots ,a_{n-1}\right)}^{T}$ and where $b={\left(b_{0},b_{1},\ldots ,b_{n-1}\right)}^{T}.$ If we apply σ to the power 3, we have

$$\sigma^{\left(3\right)}\left(\pi_{n}\left(a\left(t\right)\right)\right)=\left[\begin{array}{cc} a & b \end{array}\right]\left[\begin{array}{cccccc} 0 & 1 & 0 & 0 & 1 & 0\\ 0 & 0 & 0 & \lambda_{1} & \lambda_{2} & 1 \end{array}\right].$$

Assume there is g(t)∈I=〈a(t)+ub(t)〉 such that $\sigma^{\left(3\right)}\left(\pi_{n}\left(f\left(t\right)\right)\right)=\pi_{n}\left(g\left(t\right)\right).$ Since g(t) can be expressed as $b\left(t\right)=f_{1}\left(t\right)\left(a\left(t\right)+ub\left(t\right)\right)$ for some $f_{1}\left(t\right)\in ℜ\left(F_{q},n\right).$ The matrix associated with $\pi_{n}\left(g\left(t\right)\right)$ is of the form

$$\pi_{n}\left(g\left(t\right)\right)=\left[\begin{array}{cc} {\left(f_{1}b\right)}_{0} & {\left(f_{1}a\right)}_{0}\\ {\left(f_{1}b\right)}_{1} & {\left(f_{1}a\right)}_{1}\\ \vdots & \vdots \\ {\left(f_{1}b\right)}_{n-1} & {\left(f_{1}a\right)}_{n-1} \end{array}\right]\left[\begin{array}{cccccc} \lambda_{1} & \lambda_{2} & 1 & 0 & 0 & 0\\ 0 & 1 & 0 & 0 & 1 & 0 \end{array}\right].$$

Comparing the entries of the last columns of both matrices, we find that b(t)=0. This yields that ${\left(f_{1}g\right)}_{i}=0$ for all i, then

$$\pi_{n}\left(f\left(t\right)\right)=a\left[\begin{array}{cccccc} 0 & 1 & 0 & 0 & 1 & 0 \end{array}\right],$$

where $a={\left(a_{0},a_{1},\ldots ,a_{n-1}\right)}^{T}.$ For the converse, let $f\left(t\right)=a\left(t\right)(f_{1}\left(t\right)+uf_{2}\left(t\right)+vf_{3}\left(t\right))\in I$, which is generated by a(t), where $f_{i}\left(t\right)\in ℜ\left(F_{q},n\right).$ Thus, we have

(35) $$\pi_{n}(f(t))={\left[\lambda_{1}{\left(af_{2}\right)}_{i},{\left(af_{1}\right)}_{i}+\lambda_{2}{\left(af_{2}\right)}_{i},{\left(af_{2}\right)}_{i},\lambda_{1}{\left(af_{3}\right)}_{i},{\left(af_{1}\right)}_{i}+\lambda_{2}{\left(af_{3}\right)}_{i},{\left(af_{3}\right)}_{i}\right]}_{i=0}^{n-1}.$$

Hence,

$$\begin{array}{cc} \sigma^{\left(3\right)}\;\left(\pi_{n}\left(f\left(t\right)\right)\right)=[ & \lambda_{1}{\left(af_{3}\right)}_{i-1},\;{\left(af_{1}\right)}_{i-1}+\lambda_{2}{\left(af_{3}\right)}_{i-1},\;{\left(af_{3}\right)}_{i-1},\\ & \lambda_{1}{\left(af_{2}\right)}_{i},\;{\left(af_{1}\right)}_{i}+\lambda_{2}{\left(af_{2}\right)}_{i},\;{\left(af_{2}\right)}_{i}{]}_{i=0}^{n-1}. \end{array}$$

Choose g(t)∈I to be of the form $g\left(t\right)=a\left(t\right)(f_{1}\left(t\right)+uf_{3}\left(t\right)+vf_{2}\left(t\right)),$ where $f_{i}\left(t\right)$ is the same as that of f(t). One can see that $\sigma^{\left(3\right)}\left(\pi_{n}\left(f(t)\right)\right)=\pi_{n}\left(g\left(t\right)\right),$ then $\pi_{n}\left(C\right)$ is with index of 3. □

**Corollary** **1.** *Let C be a cyclic code of length n. Then, $\pi_{n}\left(C\right)$ is either cyclic or has index 3 or 6.*

**Proof.**  Suppose that l≥2. If l∈{2,4,5}, then one can prove that C is cyclic (the proof follows by applying the argument used in the proof of Theorem 3, and is thereby omitted). Hence, if $\pi_{n}\left(C\right)$ is not cyclic, then $\pi_{n}\left(C\right)$ has index 3 if l=3, and $\pi_{n}\left(C\right)$ has index 6 if its index is neither 1 nor 3. □

The aforementioned results offer a thorough examination of the algebraic framework of cyclic codes within the ring $R_{p,u,v}$ and their associated Gray pictures. They provide a comprehensive characterization of the potential indices for the Gray pictures of these codes. This characterization elucidates the relationship between the structural qualities of cyclic codes and the behavior of their Gray images, while also delineating specific criteria for identifying when the Gray image is cyclic or quasi-cyclic of index 3 or 6. Thus, these findings provide a cohesive framework for comprehending the interaction between cyclic codes over $R_{p,u,v}$ and their Gray map representations.

**Corollary** **2.** *Let C be a cyclic code of length n over $R_{p,u,v}$ associated with the ideal I=〈a(t)+ub(t),vc(t)〉. Then, the Gray image $\pi_{n}\left(C\right)$ is a quasi-cyclic code with index l, where l∈{1,3,6} and*

(i) *l=1 if and only if C=0;*
(ii) *l=3 if and only if I=〈a(t)〉;*
(iii) *l=6 if and only if I=〈a(t)+ub(t),vc(t)〉 and cases (i)–(ii) do not hold.*

## 4. Numerical Examples

The remainder of the study focuses on elucidating the results detailed in Section 3. We present an example demonstrating the existence of cyclic codes C over $R_{p,u,v}$ for which the Gray image $\pi_{n}\left(C\right)$ constitutes a quasi-cyclic code of index 6, even in the case where c(t)=0. This example illustrates that the presence of a Gray image with index 6 does not inherently indicate that c(t) is nonzero, revealing the intricacies in the correlation between the index of the Gray image and the elements of the generating polynomials.

**Example** **1.** *Assume I=〈ub(t)〉. We will establish that the index of $\pi_{n}\left(C\right)$ is precisely 6. Observe that for any polynomial $f_{1}\left(t\right)\in ℜ\left(F_{q},n\right)$, the following identity holds:*

$$\left(1+t+\cdots +t^{\;n-1}\right)f_{1}\left(t\right)\;=\;f_{1}\left(1\right)\;\left(1+t+\cdots +t^{\;n-1}\right).$$

*Consequently, if f(t)∈I, then by Lemma 2 we obtain*

$$f\left(t\right)\;=\;u\;f_{1}\left(1\right)\;b\left(t\right).$$

*Thus,*

$$\pi_{n}\left(f\left(t\right)\right)=\left[\begin{array}{c} {\left(f_{1}\left(1\right)b\right)}_{0}\\ \vdots \\ {\left(f_{1}\left(1\right)b\right)}_{n-1} \end{array}\right]\left[\begin{array}{cccccc} \lambda_{1} & \lambda_{2} & 1 & 0 & 0 & 0 \end{array}\right].$$

*If we set b(t)=a(t), and hence, by the definition of σ, we have*

$$\sigma \left(\pi_{n}\left(a\left(t\right)\right)\right)=\left[\begin{array}{c} {\left(f_{1}\left(1\right)b\right)}_{0}\\ \vdots \\ {\left(f_{1}\left(1\right)b\right)}_{n-1} \end{array}\right]\left[\begin{array}{cccccc} 0 & \lambda_{1} & \lambda_{2} & 1 & 0 & 0 \end{array}\right].$$

*By repeatedly applying this process six times, we obtain*

(36) $$\pi_{n}\left(g\left(t\right)\right)\;=\;\pi_{n}\left(f\left(t\right)\right)\;=\;\sigma^{6}\;\left(\pi_{n}\left(f\left(t\right)\right)\right).$$

*Moreover, for any l≤5, we have*

$$\sigma^{l}\;\left(\pi_{n}\left(f\left(t\right)\right)\right)\;\neq \;\pi_{n}\left(f\left(t\right)\right).$$

*Therefore, the index of $\pi_{n}\left(C\right)$ is exactly 6.*

In the next example, we explicitly compute the generator matrices of $\pi_{n}\left(C\right).$

**Example** **2.** *Consider the cyclic code C over the ring $R_{p,u,v}$ with parameters p=13, n=4, and m=1. The corresponding ideal of C is given by*

I=〈a(t)+ub(t),vc(t)〉,

*where the polynomials are chosen as*

$$\begin{array}{c} a\left(t\right)=\left(t-1\right)\left(t^{2}+1\right)=t^{3}-t^{2}+t-1,\;b\left(t\right)=t^{2}+1,\;c\left(t\right)=t-1. \end{array}$$

*Then, by Lemma 2, given f(t)∈I, there exist $f_{1}\left(t\right),f_{2}\left(t\right),f_{3}\left(t\right)$ and g(t) of $ℜ(F_{13},4)$ such that*

$$f\left(t\right)=a\left(t\right)f_{1}\left(t\right)+u\left(b\left(t\right)f_{1}\left(t\right)+a\left(t\right)f_{2}\left(t\right)\right)+v\left(a\left(t\right)f_{3}\left(t\right)+c\left(t\right)g\left(t\right)\right).$$

*Now, set $\lambda_{1}=\lambda_{2}=1$. Then, by Lemma 2, we have $\pi_{4}\left(f\left(t\right)\right)={\left[f_{6i+j}\right]}_{\begin{array}{c} 0\leq i\leq 3\\ 0\leq j\leq 5 \end{array}},$ where*

$$\begin{array}{cc} & f_{6i}={\left(bf_{1}\right)}_{i}+{\left(ff_{2}\right)}_{i},\;f_{6i+1}={\left(ff_{1}\right)}_{i}+{\left(bf_{1}\right)}_{i}+{\left(ff_{2}\right)}_{i},\;f_{6i+2}={\left(bf_{1}\right)}_{i}+{\left(ff_{2}\right)}_{i},\\ & f_{6i+3}={\left(ff_{3}\right)}_{i}+{\left(cg\right)}_{i},\;f_{6i+4}={\left(ff_{1}\right)}_{i}+{\left(ff_{3}\right)}_{i}+{\left(cg\right)}_{i},\;f_{6i+5}={\left(ff_{3}\right)}_{i}+{\left(cg\right)}_{i}, \end{array}$$

*for each i∈{0,…,3}. Hence, the Gray image $\pi_{4}\left(C\right)$ is a quasi-cyclic code over $F_{13}$, generated by the set of matrices $\left\{M_{i,j},N_{k}\right\}$, where i∈{1,2,3} and j,k∈{0,1,2,3}. By applying the results from Section 3, one can verify that $\pi_{4}\left(C\right)$ has length 24 and is a quasi-cyclic code of index 6.*

*Let us write*

$$f_{i}\left(t\right)=f_{i,0}+f_{i,1}t+f_{i,2}t^{2}+f_{i,3}t^{3}\;\mathrm{for}\;\mathrm{each}\;i\in \left\{1,2,3\right\},$$

*and*

$$g\left(t\right)=g_{1,0}+g_{1,1}t+g_{1,2}t^{2}+g_{1,3}t^{3},$$

*where $f_{i,j}$ and $g_{1,k}$ are elements of $F_{13}$ for j,k∈{0,1,2,3}. With this notation, the matrices $M_{i,j}$ and $N_{k}$ can be explicitly constructed from the coefficients of $f_{i}\left(t\right)$ and g(t), providing a full generating set for $\pi_{4}\left(C\right)$,*

(37) $$\pi_{4}\left(f\left(t\right)\right)=\sum_{i\in \{1,2,3\},j\in \{0,1,2,3\}}f_{i,j}G_{i,j}+\sum_{k\in \{0,1,2,3\}}g_{1,k}H_{k}.$$

*Here are all $G_{i,j}$ and $H_{k}$ matrices for p=13:*

*1.*  *Matrices $G_{1,j}$ associated with $f_{1}\left(t\right)$:*
$$G_{1,0}=\left(\begin{array}{cccccc} 1 & 12 & 1 & 0 & 0 & 0\\ 0 & 1 & 0 & 0 & 0 & 0\\ 1 & 12 & 1 & 0 & 0 & 0\\ 0 & 0 & 0 & 0 & 0 & 0 \end{array}\right),\;\;\;G_{1,1}=\left(\begin{array}{cccccc} 0 & 1 & 0 & 0 & 0 & 0\\ 1 & 12 & 1 & 0 & 0 & 0\\ 0 & 1 & 0 & 0 & 0 & 0\\ 1 & 12 & 1 & 0 & 0 & 0 \end{array}\right)$$
$$G_{1,2}=\left(\begin{array}{cccccc} 1 & 12 & 1 & 0 & 0 & 0\\ 0 & 1 & 0 & 0 & 0 & 0\\ 1 & 12 & 1 & 0 & 0 & 0\\ 0 & 0 & 0 & 0 & 0 & 0 \end{array}\right),\;\;\;G_{1,3}=\left(\begin{array}{cccccc} 0 & 1 & 0 & 0 & 0 & 0\\ 1 & 12 & 1 & 0 & 0 & 0\\ 0 & 1 & 0 & 0 & 0 & 0\\ 1 & 12 & 1 & 0 & 0 & 0 \end{array}\right)$$
*2.*  *Matrices $G_{2,j}$ associated with $f_{2}\left(t\right)$:*
$$G_{2,0}=\left(\begin{array}{cccccc} 12 & 12 & 12 & 0 & 0 & 0\\ 1 & 1 & 1 & 0 & 0 & 0\\ 12 & 12 & 12 & 0 & 0 & 0\\ 1 & 1 & 1 & 0 & 0 & 0 \end{array}\right),\;\;\;G_{2,1}=\left(\begin{array}{cccccc} 1 & 1 & 1 & 0 & 0 & 0\\ 12 & 12 & 12 & 0 & 0 & 0\\ 1 & 1 & 1 & 0 & 0 & 0\\ 12 & 12 & 12 & 0 & 0 & 0 \end{array}\right)$$
$$G_{2,2}=\left(\begin{array}{cccccc} 12 & 12 & 12 & 0 & 0 & 0\\ 1 & 1 & 1 & 0 & 0 & 0\\ 12 & 12 & 12 & 0 & 0 & 0\\ 1 & 1 & 1 & 0 & 0 & 0 \end{array}\right),\;\;\;G_{2,3}=\left(\begin{array}{cccccc} 1 & 1 & 1 & 0 & 0 & 0\\ 12 & 12 & 12 & 0 & 0 & 0\\ 1 & 1 & 1 & 0 & 0 & 0\\ 12 & 12 & 12 & 0 & 0 & 0 \end{array}\right)$$
*3.*  *Matrices $G_{3,j}$ associated with $f_{3}\left(t\right)$:*
$$G_{3,0}=\left(\begin{array}{cccccc} 0 & 0 & 0 & 12 & 12 & 12\\ 0 & 0 & 0 & 1 & 1 & 1\\ 0 & 0 & 0 & 12 & 12 & 12\\ 0 & 0 & 0 & 1 & 1 & 1 \end{array}\right),\;\;\;G_{3,1}=\left(\begin{array}{cccccc} 0 & 0 & 0 & 1 & 1 & 1\\ 0 & 0 & 0 & 12 & 12 & 12\\ 0 & 0 & 0 & 1 & 1 & 1\\ 0 & 0 & 0 & 12 & 12 & 12 \end{array}\right)$$
$$G_{3,2}=\left(\begin{array}{cccccc} 0 & 0 & 0 & 12 & 12 & 12\\ 0 & 0 & 0 & 1 & 1 & 1\\ 0 & 0 & 0 & 12 & 12 & 12\\ 0 & 0 & 0 & 1 & 1 & 1 \end{array}\right),\;\;\;G_{3,3}=\left(\begin{array}{cccccc} 0 & 0 & 0 & 1 & 1 & 1\\ 0 & 0 & 0 & 12 & 12 & 12\\ 0 & 0 & 0 & 1 & 1 & 1\\ 0 & 0 & 0 & 12 & 12 & 12 \end{array}\right)$$
*4.*  *Matrices $H_{k}$ associated with g(t):*
$$H_{0}=\left(\begin{array}{cccccc} 0 & 0 & 0 & 12 & 12 & 12\\ 0 & 0 & 0 & 1 & 1 & 1\\ 0 & 0 & 0 & 0 & 0 & 0\\ 0 & 0 & 0 & 0 & 0 & 0 \end{array}\right),\;\;\;H_{1}=\left(\begin{array}{cccccc} 0 & 0 & 0 & 0 & 0 & 0\\ 0 & 0 & 0 & 12 & 12 & 12\\ 0 & 0 & 0 & 1 & 1 & 1\\ 0 & 0 & 0 & 0 & 0 & 0 \end{array}\right)$$
$$H_{2}=\left(\begin{array}{cccccc} 0 & 0 & 0 & 0 & 0 & 0\\ 0 & 0 & 0 & 0 & 0 & 0\\ 0 & 0 & 0 & 12 & 12 & 12\\ 0 & 0 & 0 & 1 & 1 & 1 \end{array}\right),\;\;\;H_{3}=\left(\begin{array}{cccccc} 0 & 0 & 0 & 1 & 1 & 1\\ 0 & 0 & 0 & 0 & 0 & 0\\ 0 & 0 & 0 & 0 & 0 & 0\\ 0 & 0 & 0 & 12 & 12 & 12 \end{array}\right)$$

**Example** **3.** *Let p=3,n=4 and m=2, then $R_{p,u,v}=F_{9}+uF_{9}+vF_{9}.$ Suppose that $F_{9}=\frac{F_{3}\left[\alpha \right]}{\langle \alpha^{2}+1\rangle },$ where $\alpha^{2}=2$. Moreover, we assume*

$$a\left(t\right)=t^{3}-t^{2}+t-1,\;b\left(t\right)=t^{2}+1,\;c\left(t\right)=t-1.$$

*For $f\left(t\right)=a\left(t\right)g_{1}\left(t\right)+u\left(b\left(t\right)g_{1}\left(t\right)+a\left(t\right)g_{2}\right)+v(a\left(t\right)g_{3}\left(t\right)+c\left(t\right)h_{1}\left(t\right)),$ then,*

(38) $$\pi_{4}\left(f\left(t\right)\right)={\left[f_{6i+j}\right]}_{\begin{array}{c} 0\leq i\leq 3\\ 0\leq j\leq 5 \end{array}},$$

*where the associated relations $f_{i,j}=f_{6i+j}$ are of the form*

$$\begin{array}{cc} f_{i,0} & ={\left(f_{2}g_{1}\right)}_{i}+{\left(f_{1}g_{2}\right)}_{i},\;f_{i,1}={\left(f_{1}g_{1}\right)}_{i}+{\left(f_{2}g_{1}\right)}_{i}+{\left(f_{1}g_{2}\right)}_{i},\\ f_{i,2} & ={\left(f_{2}g_{1}\right)}_{i}+{\left(f_{1}g_{2}\right)}_{i},\;f_{i,3}={\left(f_{1}g_{3}\right)}_{i}+{\left(a_{2}h_{1}\right)}_{i},\\ f_{i,4} & ={\left(f_{1}g_{1}\right)}_{i}+{\left(f_{1}g_{3}\right)}_{i}+{\left(a_{2}h_{1}\right)}_{i},\;f_{i,5}={\left(f_{1}g_{3}\right)}_{i}+{\left(a_{2}h_{1}\right)}_{i}. \end{array}$$

*The associated matrices are of the form*

$$G_{1,0}=\left(\begin{array}{cccccc} 1 & 0 & \alpha & 0 & 0 & 0\\ 0 & 1 & 0 & 0 & 0 & 0\\ \alpha & 0 & 1 & 0 & 0 & 0\\ 0 & 1 & 0 & 0 & 0 & 0 \end{array}\right),\;G_{1,1}=\left(\begin{array}{cccccc} 0 & 1 & 0 & 0 & 0 & 0\\ 1 & 0 & \alpha & 0 & 0 & 0\\ 0 & 1 & 0 & 0 & 0 & 0\\ \alpha & 0 & 1 & 0 & 0 & 0 \end{array}\right)$$

$$G_{1,2}=\left(\begin{array}{cccccc} 1 & 0 & \alpha & 0 & 0 & 0\\ 0 & 1 & 0 & 0 & 0 & 0\\ \alpha & 0 & 1 & 0 & 0 & 0\\ 0 & 1 & 0 & 0 & 0 & 0 \end{array}\right),\;G_{1,3}=\left(\begin{array}{cccccc} 0 & 1 & 0 & 0 & 0 & 0\\ 1 & 0 & \alpha & 0 & 0 & 0\\ 0 & 1 & 0 & 0 & 0 & 0\\ \alpha & 0 & 1 & 0 & 0 & 0 \end{array}\right)$$

$$G_{2,0}=\left(\begin{array}{cccccc} 2 & 2\alpha & 2 & 0 & 0 & 0\\ 1 & \alpha & 1 & 0 & 0 & 0\\ 2 & 2\alpha & 2 & 0 & 0 & 0\\ 1 & \alpha & 1 & 0 & 0 & 0 \end{array}\right),\;G_{2,1}=\left(\begin{array}{cccccc} 1 & \alpha & 1 & 0 & 0 & 0\\ 2 & 2\alpha & 2 & 0 & 0 & 0\\ 1 & \alpha & 1 & 0 & 0 & 0\\ 2 & 2\alpha & 2 & 0 & 0 & 0 \end{array}\right)$$

$$G_{2,2}=\left(\begin{array}{cccccc} 2 & 2\alpha & 2 & 0 & 0 & 0\\ 1 & \alpha & 1 & 0 & 0 & 0\\ 2 & 2\alpha & 2 & 0 & 0 & 0\\ 1 & \alpha & 1 & 0 & 0 & 0 \end{array}\right),\;G_{2,3}=\left(\begin{array}{cccccc} 1 & \alpha & 1 & 0 & 0 & 0\\ 2 & 2\alpha & 2 & 0 & 0 & 0\\ 1 & \alpha & 1 & 0 & 0 & 0\\ 2 & 2\alpha & 2 & 0 & 0 & 0 \end{array}\right)$$

$$G_{3,0}=\left(\begin{array}{cccccc} 0 & 0 & 0 & 2 & 2\alpha & 2\\ 0 & 0 & 0 & 1 & \alpha & 1\\ 0 & 0 & 0 & 2 & 2\alpha & 2\\ 0 & 0 & 0 & 1 & \alpha & 1 \end{array}\right),\;G_{3,1}=\left(\begin{array}{cccccc} 0 & 0 & 0 & 1 & \alpha & 1\\ 0 & 0 & 0 & 2 & 2\alpha & 2\\ 0 & 0 & 0 & 1 & \alpha & 1\\ 0 & 0 & 0 & 2 & 2\alpha & 2 \end{array}\right)$$

$$G_{3,2}=\left(\begin{array}{cccccc} 0 & 0 & 0 & 2 & 2\alpha & 2\\ 0 & 0 & 0 & 1 & \alpha & 1\\ 0 & 0 & 0 & 2 & 2\alpha & 2\\ 0 & 0 & 0 & 1 & \alpha & 1 \end{array}\right),\;G_{3,3}=\left(\begin{array}{cccccc} 0 & 0 & 0 & 1 & \alpha & 1\\ 0 & 0 & 0 & 2 & 2\alpha & 2\\ 0 & 0 & 0 & 1 & \alpha & 1\\ 0 & 0 & 0 & 2 & 2\alpha & 2 \end{array}\right)$$

$$H_{0}=\left(\begin{array}{cccccc} 0 & 0 & 0 & 2 & 2\alpha & 2\\ 0 & 0 & 0 & 1 & \alpha & 1\\ 0 & 0 & 0 & 0 & 0 & 0\\ 0 & 0 & 0 & 0 & 0 & 0 \end{array}\right),\;H_{1}=\left(\begin{array}{cccccc} 0 & 0 & 0 & 0 & 0 & 0\\ 0 & 0 & 0 & 2 & 2\alpha & 2\\ 0 & 0 & 0 & 1 & \alpha & 1\\ 0 & 0 & 0 & 0 & 0 & 0 \end{array}\right)$$

$$H_{2}=\left(\begin{array}{cccccc} 0 & 0 & 0 & 0 & 0 & 0\\ 0 & 0 & 0 & 0 & 0 & 0\\ 0 & 0 & 0 & 2 & 2\alpha & 2\\ 0 & 0 & 0 & 1 & \alpha & 1 \end{array}\right),\;H_{3}=\left(\begin{array}{cccccc} 0 & 0 & 0 & 1 & \alpha & 1\\ 0 & 0 & 0 & 0 & 0 & 0\\ 0 & 0 & 0 & 0 & 0 & 0\\ 0 & 0 & 0 & 2 & 2\alpha & 2 \end{array}\right)$$

## 5. Conclusions

This study has analyzed the Gray images of cyclic and self-orthogonal codes of length *n* over the local non-Frobenius ring

(39) $$R_{p,u,v}=F_{q}+uF_{q}+vF_{q},$$

with the conditions that $u^{2}=v^{2}=uv=vu=0$. We presented the Gray map $\pi_{n}:R_{p,u,v}^{n}\to F_{q}^{6n}$ and established that, given the conditions gcd(n,p)=1 and p∉{2,5}, every cyclic code C over $R_{p,u,v}$ of length *n* is transformed into a quasi-cyclic code of length 6n over $F_{q}$ with index l≤6. We demonstrated that the index of $\pi_{n}\left(C\right)$ is restricted to the values 1,3, or 6, and we established the necessary and sufficient conditions for each scenario. The findings offer valuable insights into the structural behavior of codes over non-chain rings and emphasize the significance of Gray maps in connecting the study of ring-based codes with quasi-cyclic codes over finite fields. Furthermore, the incorporation of numerical examples has demonstrated the practical significance of our theoretical findings.

This work presents opportunities for additional research, including the examination of other classes of codes over analogous non-chain rings, broadening the analysis to various Gray mappings, and exploring potential applications within coding theory and cryptography. In addition to the structural results obtained here, a systematic investigation of the parameters of the Gray images, particularly their minimum distance and optimality properties, would be of interest. Moreover, a complete study of self-dual cyclic codes over $R_{p,u,v}$ and the behavior of their Gray images constitutes a natural extension of the present work.

## Data Availability

The original contributions presented in this study are included in the article. Further inquiries can be directed to the corresponding author.
